# Symptoms and impacts of familial chylomicronemia syndrome: a qualitative study of the patient experience

**DOI:** 10.1186/s13023-023-02927-8

**Published:** 2023-10-11

**Authors:** Kate Williams, Georgina Tickler, Pedro Valdivielso, Jordi Alonso, Montserrat Vera-Llonch, Laia Cubells, Sarah Acaster

**Affiliations:** 1 Acaster Lloyd Consulting, London, UK; 2 Servicio de Medicina Interna, Hospital Virgen de la Victoria, University of Málaga and Instituto de Investigaciones Biomédicas de Málaga (IBIMA), Málaga, Spain; 3 IMIM-Institut Hospital del Mar d’Investigacions Mèdiques, CIBERESP, Pompeu Fabra University, Barcelona, Spain; 4 Ionis Pharmaceuticals, One Beacon Street, Boston, MA 02120 USA; 5 Akcea Therapeutics, Avenida Ernest Lluch, 32 TCM 2 of 6.18, 08302 Mataró, Barcelona, Spain

**Keywords:** Familial chylomicronemia syndrome, FCS, Qualitative, Symptoms, Health-related quality of life, Conceptual model

## Abstract

**Background:**

Familial chylomicronemia syndrome (FCS) is a rare, hereditary, metabolic disorder. FCS causes high levels of triglycerides in the blood, which can lead to abdominal pain, xanthomas, and acute pancreatitis (AP). Volanesorsen, along with adherence to a very low-fat diet is used to reduce triglyceride levels in individuals with FCS. We aimed to understand the symptoms of FCS and their impact on health-related quality of life (HRQoL).

**Methods:**

Interviews were conducted with individuals with genetically confirmed FCS in the UK and Spain, some of whom had been treated with volanesorsen. Interview guides were developed with input from a patient advocacy group to explore the symptoms, impacts and management of FCS. Interviews were conducted by telephone and were recorded and transcribed. Data were analyzed using thematic analysis and saturation was recorded.

**Results:**

Seventeen interviews were conducted with individuals with FCS (aged 27–68 years), thirteen of whom were currently/previously treated with volanesorsen. Episodes of AP were the most impactful reported symptom, resulting in severe abdominal pain, nausea, vomiting, fever, bloating and appetite loss. Other symptoms and functional issues included abdominal pain, gastrointestinal symptoms, impaired cognitive function and fatigue. These had an impact on work, social activities, relationships and psychological wellbeing. These symptoms and impacts were illustrated in a conceptual model, including management strategies. The challenges of managing a low-fat diet and experience with volanesorsen were discussed.

**Conclusion:**

Individuals with FCS experience a range of interrelated symptoms and functional limitations which impact their broader HRQoL. Treatments which alleviate symptoms and reduce the incidence of AP episodes have the potential to improve the HRQoL of these individuals.

## Introduction

Familial chylomicronemia syndrome (FCS) is a rare, hereditary, metabolic disorder with an estimated global prevalence of 1 to 2 per 1,000,000 [1, 2]. FCS is characterized by severe hypertriglyceridemia secondary to the accumulation of chylomicrons and very-low density lipoprotein in plasma due to deficient lipolytic activity [1], which leads to symptoms including abdominal pain, eruptive xanthomas, and acute pancreatitis (AP) [3]. The deficient lipolytic activity that defines FCS is mostly caused from mutations in the *LPL* gene, however mutations in *GPIHBP1*, *LMF1*, *APOA5* and *APOC2* genes have also been identified to be causative of the disease [4].

The long-term management of FCS relies on adherence to healthy lifestyle changes, including following an extremely restrictive, low-fat diet (10–20 g per day), limiting sugar intake, eating small frequent meals, taking supplements and minerals, and life-long avoidance of alcohol and medications known to increase triglyceride levels [4, 5]. However, adherence to these strict requirements is often challenging and does not always prevent the onset of acute pancreatitis [5]. In addition, conventional pharmacological treatments (e.g., omega-3 fatty acids, fibrates and statins) that aim to reduce triglyceride levels have had typically limited success in individuals with FCS [6–8]. A promising therapeutic target in FCS is the amino acid protein, ApoC-III, which interacts with chylomicrons and other lipoproteins. ApoC-III interacts with lipoprotein metabolism, including the secretion of TG-rich lipoproteins, and interferes with LPL activity and obstructs the clearance of TG-rich lipoproteins [9]. Volanesorsen is the only licensed pharmacological treatment for FCS in the European Union and UK [10]. It is an antisense drug that binds to hepatic *APOC3* mRNA, leading to its degradation and preventing the translation of the ApoC-III protein. Volanesorsen aims to reduce the level of triglycerides (chylomicrons) in the blood and decrease the risk of acute pancreatitis. Individuals with FCS may also take medications to control pain and other symptoms, for example, antithrombotic, analgesics and antidepressants [11].

A recent narrative review highlighted that a very low-fat diet continues to be central to the management of FCS [12]. In a multi-country survey of 166 individuals with FCS, 93% reported that they struggled with managing their fat intake and 53% still experienced symptoms despite adherence to their diets [3]. More than two thirds of participants reported that their emotional wellbeing was significantly affected by FCS and 75% reported that their social lives were restricted. This survey also found that 94% of those who were unemployed or employed part-time attributed their employment status to FCS [3].

Qualitative research provides the opportunity to hear about the patient’s experience in their own words. A recent qualitative interview study was conducted with 10 individuals with FCS to identify concepts to include in a new patient-reported outcome (PRO) measure [13]. This identified a range of important symptoms, including abdominal pain, physical fatigue, difficulty thinking and diarrhea, as well as a range of impacts. To-date, no qualitative studies have explored the impact of the restrictive diet, to which all individuals with FCS are advised to adhere, nor examined experiences with treatments for FCS, such as volanesorsen. The primary aim of this study was to explore the symptoms and impacts of FCS, as well as the experience of managing FCS through adherence to a low-fat diet and treatment with volanesorsen. A second aim was to develop a conceptual model outlining the relationships between these symptoms, impacts and challenges.

## Materials and methods

### Design and participants

Qualitative interviews were conducted with individuals with FCS, some of whom had been treated with volanesorsen in the United Kingdom and Spain. Inclusion criteria were: (i) Genetically confirmed diagnosis of FCS, (ii) Aged 18 + years, (iii) Resident of the UK or Spain, (iv) Good level of spoken English or Spanish, sufficient to take part in an interview, (v) Willing and able to provide consent to take part in a one-hour interview. In addition, (vi) Those who were currently on volanesorsen were required to have been on treatment for at least six months, while (vii) Those previously treated with volanesorsen could have been on volanesorsen for any length of time.

### Study materials

Three separate semi-structured interview guides were developed for individuals with FCS: (1) currently on volanesorsen, (2) previously on volanesorsen, (3) never on volanesorsen. The content of the interview guides was based on a targeted review of the published literature on the symptoms and impacts of FCS and through consultation with a patient representative from the Lipoprotein Lipase Deficiency (LPLD) Alliance (now Action FCS). The interview guides comprised mainly of open-ended questions on the symptoms of FCS, as well as its impacts on daily life and HRQoL, as well as experience of managing their condition. Participants were asked about their current experience managing and treating their FCS. Those who had been treated with volanesorsen were asked questions in relation to their experience both before and while they were on treatment, and those who had stopped treatment were also asked about their experience since stopping.

Three background questionnaires were also developed to collect socio-demographic information, as well as information on their FCS diagnosis and symptoms, treatment and diet. Participants were also asked to complete the newly developed FCS Symptoms and Impacts Scale (FCS-SIS), a 17-item PRO instrument [13]. This includes four symptom questions, which ask about abdominal pain, physical fatigue, difficulty thinking and diarrhea, on an 11-point numeric rating scale from 0 = no symptom/difficulty to 10 = worst possible symptom/difficulty, with a 24-h recall, and 13 impact questions (e.g. ‘I worry about having a pancreatitis attack’), which are scored on a 5-point Likert scale from never to always. All study materials were developed in English and translated into Spanish.

### Ethics review and approval

The UK study was reviewed and approved by the WIRB-Copernicus Group Independent Review Board (IRB tracking number: #20200269). In Spain, the study was reviewed and approved on 24th September 2020 by the Comité de Ética de la Investigación Provincial de Málaga.

### Recruitment and interviews

UK participants were recruited by Action FCS using purposive sampling. Potential participants were contacted by email and were asked to contact the research team if they were interested. Participants in Spain were recruited by clinical sites. Clinicians identified eligible patients and invited them to participate either during their regular visits or by phone/letter. Participant eligibility was confirmed using a screening questionnaire based on the inclusion criteria. Due to the limited pool of potentially eligible participants, all of those who were eligible were included and no additional sampling criteria were used. Participants were sent a background questionnaire and the FCS-SIS, to complete and return by email, as well as an information and consent form to read in advance. Participants in Spain returned a signed consent to their clinician prior to taking part. Participants in both countries provided verbal informed consent at the start of their interview.

UK interviews were conducted by two qualitative researchers, both with postgraduate degrees in psychology (KW and NP). The interviews in Spain were conducted by qualitative interviewers who were native Spanish speakers but who also spoke English. The interviews were conducted by telephone/Zoom between March 2020 and May 2021. Interviews followed the relevant semi-structured interview guide, lasted around one hour, and were recorded. Participants were informed that they could refuse to answer any questions they did not wish to answer and were given the opportunity to speak freely. If participants answered inconsistently, they were probed to clarify any ambiguities.

The interview recordings were transcribed, and the transcripts were de-identified using participant identification numbers and names were removed prior to analysis. These were stored on a secure server, separate from any participant names and contact details. Any adverse events were reported in accordance with pharmacovigilance procedures.

### Analyses

Data from the background questionnaire were summarized using descriptive statistics. Data from the interviews were analyzed using thematic analysis in MAXQDA, a software package that facilitates analysis but does not automate any of the process [14]. All researchers involved in the analysis had postgraduate degrees in psychology. Two researchers first read through all the UK transcripts and developed an initial coding framework based on the topics covered in the interview guide (KW and NP). They then coded one transcript (participant number: 101) independently and compared notes and discussed discrepancies. The initial coding framework was then revised following this discussion and one researcher (NP) coded the remaining UK transcripts (including re-coding the original transcript) using the updated framework. The Spanish transcripts were translated into English for analysis. The Spanish data were analyzed by two different researchers (GT and HdF), after each transcript was coded, the researchers discussed discrepancies and made additional amendments to the coding framework as needed. When codes were added or changed, the previously coded transcripts were reviewed, and the new codes were applied as needed. A final quality review of all transcripts was conducted by KW. The codes were then reviewed in detail to identify relevant concepts (symptoms and impacts), which were then grouped into themes to describe the experience of living with FCS.

Rather than target a specific sample size, best practice in qualitative research is to keep conducting interviews until data saturation is reached. Data saturation has been defined as the point at which no new insights are obtained, or no new themes are identified in the data [15, 16]. A saturation matrix was used to monitor the frequency of reported concepts across the interviews, where the concepts were listed in rows and the interviews were listed in columns in order of completion [17].

A conceptual model was developed to illustrate the symptoms and impacts, as well as the management strategies for dealing with these. The symptoms and impacts were described in boxes and arrows were used to indicate the direction of the relationships between these symptoms and impacts. These relationships were based solely on the qualitative data. The symptoms and impacts of FCS reported here describe the experiences of participants before taking volanesorsen and/or the experiences of individuals who had never taken volanesorsen.

## Results

### Sample characteristics

Seventeen individuals with FCS took part in the interviews. Socio-demographic characteristics are shown in Table 1. Nine participants were employed part or full time, while five were retired, unemployed or on long-term sick leave. Four participants had higher education below degree level and five participants had a university degree or higher. Clinical characteristics of the sample are shown in Table 2. Five individuals had been previously diagnosed with diabetes, representing 29.4% of the participants. Six individuals were currently on volanesorsen, seven had previously been treated and four had never been treated with volanesorsen. Of those individuals currently on volanesorsen, the median time since starting treatment was 2.58 years. For those that had previously taken the drug and had stopped, the median time on volanesorsen was 0.83 years. When assessing episodes of acute pancreatitis (AP), the median number of years since the last AP episode was two years and the median number of episodes in the past five years was also two. Finally, for individuals currently on volanesorsen, none had experienced an episode of AP since starting the drug.

**Table 1:** Socio-demographic characteristics (N = 17)

Characteristic	Median (IQR)
Current age (years)	48.0 (45–56)
Age when noticed first symptoms (years)	7.0 (4–13)
Age at diagnosis (years)	25.0 (12–40)
*Sex*	**N (%)**
Male	2 (12)
Female	15 (88)
*Ethnic background*	
White	13 (76.5)
Asian or Asian British	4 (23.5)
*Education*	
No formal qualifications	1 (5.8)
ONC/BTEC	2 (11.8)
O level/GSCE or equivalent	2 (11.8)
A Level or Higher	2 (11.8)
Higher education below degree level	4 (23.5)
University degree or higher	5 (29.4)
Other	1 (5.8)
*Employment*	
Employed full-time	5 (29.4)
Employed part-time	4 (23.5)
Self employed	2 (11.8)
Full-time homemaker/caregiver	1 (5.8)
Retired	2 (11.8)
Seeking work/Unemployed	2 (11.8)
Long term sick leave	1 (5.8)

*A Level* Advanced Level. *BTEC* Business and Technology Education Council. *GCSE* General Certificate of Secondary Education. *O Level* Ordinary Level. *ONC* Ordinary National Certificate. *IQR* Interquartile range. Data are shown as median (IQR) or N(%)

**Table 2:** Clinical characteristics

Characteristic	N (%)
*Diagnosis of diabetes*	
Yes	5 (29.4)
No	12 (70.6)
*Ever taken volanesorsen*	
Currently taking	6 (35.3)
Previously taken	7 (41.2)
Never	4 (23.5)
*Time on volanesorsen*	**Median (IQR)**
Time since starting treatment with volanesorsen (years)^1^ (N = 6)	2.58 (1.77–3.15)
Time on volanesorsen before stopping (years) (N = 7)	0.83 (0.46–1.58)
*Episodes of acute pancreatitis*	
Time since last AP episode (years) (N = 15)^2^	2.00 (0.58–6.33)
Number of episodes in the last 5 years (N = 15)^2^	2.00 (0–4.5)
Number of episodes since on volanesorsen (N = 6)	0.0 (0–0.75)

^1^Those currently on volanesorsen.

^2^Response of two participants were missing. *IQR* Interquartile range. Data are shown as median (IQR) or N (%)

Summary responses to the FCS-SIS are shown in Table 3.

**Table 3:** FCS symptoms and impacts scale summary responses (N = 17)

Question	Median (IQR)	N (%)					
Symptoms (0 = no to 10 = worst possible)		0	1–2	3–4	5–6	7–8	9–10
Abdominal pain	0.0 (0–5)	10 (58.8)	2 (11.8)	0 (0)	3 (17.7)	1 (5.9)	1 (5.9)
Physical fatigue	5.0 (1–6)	4 (23.5)	3 (17.7)	1 (5.9)	6 (35.3)	2 (11.8)	1 (5.9)
Difficulty thinking	2.0 (0–5)	6 (35.3)	5 (29.4)	1 (5.9)	4 (23.5)	0 (0)	1 (5.9)
Diarrhea	0.0 (0–2)	12 (70.6)	2 (11.8)	2 (11.8)	1 (5.9)	0 (0)	0 (0)

Impacts (0 = never, 1 = rarely, 2 = sometimes, 3 = often, 4 = always)		0	1	2	3	4	–
Worry about pancreatitis attack	3.0 (3–4)	0 (0)	2 (11.8)	2 (11.8)	5 (29.4)	8 (47.1)	–
Avoid making plans	2.0 (1–3)	3 (17.7)	4 (23.5)	5 (29.4)	3 (17.7)	2 (11.8)	–
Feel anxious in social situations involving food	3.0 (2–4)	2 (11.8)	0 (0)	3 (17.7)	5 (29.4)	7 (41.2)	–
Avoid social situations involving food	3.0 (2–3)	2 (11.8)	1 (5.9)	4 (23.5)	6 (35.3)	4 (23.5)	–
Worry about eating food prepared by someone else	3.0 (2–4)	0 (0)	2 (11.8)	6 (35.3)	4 (23.5)	5 (29.4)	–
Worry about going over dietary fat limit	3.0 (3–4)	0 (0)	2 (11.8)	2 (11.8)	6 (35.3)	7 (41.2)	–
Worry about future health	4.0 (3–4)	0 (0)	0 (0)	1 (5.9)	4 (23.5)	12 (70.1)	–
Worry about being a burden	3.0 (2–4)	0 (0)	1 (5.9)	6 (35.3)	2 (11.8)	8 (47.1)	–
Less physically active	2.0 (0–4)	6 (35.3)	2 (11.8)	1 (5.9)	2 (11.8)	6 (35.3)	–
Feel sad or depressed	2.0 (1–3)	4 (23.5)	1 (5.9)	6 (35.3)	2 (11.8)	4 (23.5)	–
Feel judged by others	1.0 (0–3)	8 (47.1)	2 (11.8)	2 (11.8)	3 (17.7)	2 (11.8)	–
Worry about finances	2.0 (0–4)	5 (29.4)	2 (11.8)	4 (23.5)	0 (0)	6 (35.3)	–
Less productive	2.0 (0–3)	6 (35.3)	2 (11.8)	3 (17.7)	2 (11.8)	4 (23.5)	–

*One participant provided a range for many of their responses, so the higher number in their range was used for calculations. *IQR* Interquartile range

The saturation matrices indicated that saturation had been reached for the total sample, with all but one concept emerging in the first four interviews, and the last new concept was mentioned in the seventh interview (Table 4).

**Table 4:** Data saturation matrix

	Participant number*																
	101	102	104	106	107	108	103	109	110	111	112	113	114	116	117	118	119
*Symptoms*																	
Acute pancreatitis	**S**	S	S	S	S	S	S	S	S	S	S	S	S	S		S	S
Other abdominal pain	P	**S**	S		S	S	S	S	S			S		S	S	S	S
Other pain	P	**S**	S	S	S	S	S	S			S	S	S	S			S
GI – Nausea/vomiting		**S**	S		S	S	S	S			S	S	S	S			
GI – Diarrhea	**S**		S	S			S	S							S		S
GI – Constipation			**S**									S	S				
GI – Bloating	**S**	S	S		S			S	P		S	S	P		S	S	
GI – Indigestion				**S**							S	S	P			S	
Fatigue	P	**S**	S	S	S	S	S	S	S		S	P	S			S	P
Cognitive – Memory/brain fog				S	S	S		S	S			P	S	P	P		
Cognitive – Concentration	**S**			S			S		P			P					
*Impacts*																	
Daily activities/work	**S**	S	S	S	S	S	S	S	S				S	S	S	S	S
Social activities	**S**	S	S		S		S	S	S		S		S	S	S	S	P
Family and relationships	**S**	S	S	S	S	S	S	S	S	S	S	S	S	P	S	P	
Psychological – Anxiety/worry/stress	**S**		S	S		S	S	S				S	S	S	S	S	S
Psychological – Diagnosed depression	**S**						S	S									
Psychological – Depressive symptoms			**S**	S		S			S	S			S	P	S	P	S
Psychological – Guilt/feeling a burden			**S**			S	S										S
Psychological – Self-esteem/confidence	**S**		S	S	S												
Psychological – Loneliness							**S**		S					S			
Psychological – Appearance/self-consciousness	**S**		S	S	S			S	S								S

*GI* Gastrointestinal. *S* Spontaneously reported (bolded cell is first spontaneous report). *P* Probed. *Participant numbers are not in numerical order as they are listed here in the order the interviews were conducted

### Overview of symptoms and impacts of FCS 

**Fig. 1:**
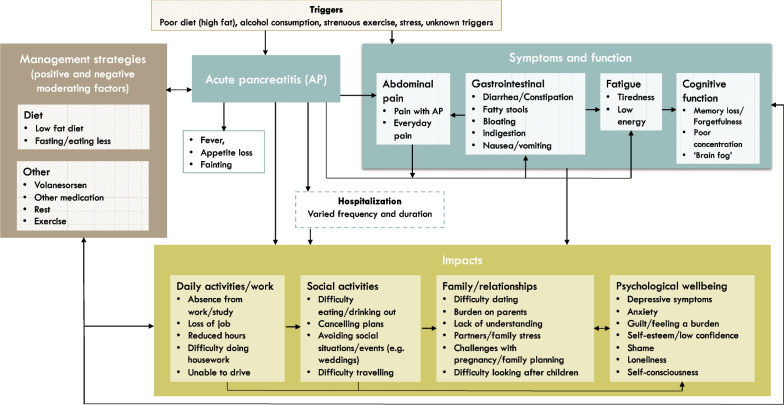
Conceptual model on the relationship between symptoms and impacts of FCS before treatment with volanesorsen

The symptoms and impacts of FCS and relationships between them are shown in a conceptual model in Fig. 1. Where applicable, this shows the symptoms and impacts when asked about the period before they started taking volanesorsen. AP, abdominal pain, gastrointestinal symptoms, fatigue and impaired cognitive function were the core symptoms and issues reported. These had an impact on daily activities and work, social activities, family life and relationships, and psychological wellbeing. A range of management strategies for managing the symptoms and impacts were reported. These are described in further detail in the sections below.

### Symptoms and function 

#### Acute pancreatitis

All but one participant reported experiencing episodes of AP before starting treatment with volanesorsen. Those who had experienced AP episodes described them as being extremely painful. Participants recalled their experiences of AP vividly and provided detailed descriptions of the severity of the pain. Some respondents described it as an explosion, a bloating or bursting pain, or a stabbing pain.

*“It was like someone was stabbing me repeatedly in the stomach. I’d be doubled over in pain, unable to concentrate, unable to sit up”* – 101 (M, 41 years, UK, currently on volanesorsen).

Participants used descriptors such as ‘horrific’, ‘horrendous’, ‘terrible’, ‘unbearable’ and ‘agony’.

*“I just wanted to go out and get hit by some vehicle because I cannot tolerate that pain and the pain is, if one person experienced that pain, they will never forget it” –* 108 (F, 45 years, UK, currently on volanesorsen).

Most participants said their episodes of AP started very suddenly as abdominal pain, although one said it started as a low pain that progressively grew worse, and another said it would come and go in waves.

Other than pain, the most commonly reported symptoms during an episode of AP were nausea and vomiting (N = 10). Two participants reported dizziness and fainting, thought to be triggered by the severe pain of an AP episode. Two participants reported having a fever or feeling hot and shaking during an AP episode.

*“…that sudden onset of epigastric pain, and just hope that it would be going away, and actually it just intensifies, and then you start to feel sick, sometimes I'd feel really sweaty with fevers as well”* − 104 (F, 32 years, UK, currently on volanesorsen).

The frequency of AP episodes varied between participants. Some reported having episodes of AP not long before they started treatment with volanesorsen, whereas others had not experienced an episode for several years. Several participants reported having episodes of AP more frequently when they were younger.

*“My life would frequently get interrupted by these pancreatic hospitalizations, they’d become quite regular. So in a year I could have you know five or six of these, it could be happening every couple of months” *− 101 (M, 41 years, UK, currently on volanesorsen).

The duration of the AP episodes also varied between participants. Most participants reported being hospitalized when they experienced an episode of AP, with stays generally lasting around three to six days (N = 7). Some said they had spent two or three weeks in hospital on certain occasions (N = 3), and two staying in hospital for a month or longer.

One participant reported that over time they had learned to manage less severe episodes of AP at home, instead of going to the hospital for treatment, and another said they had never been hospitalized for AP.

Six participants reported that their episodes of acute AP were triggered by not adhering to a low-fat diet.

*“I’d be going out on curry nights with friends, not curry houses like they are now, they were little backstreet places…I’d be eating fish and chips from the chippy. I’d be doing all sorts of, what normal people did, but then I would be paying for it” *− 102 (F, 68 years, UK, currently on volanesorsen).

One respondent in Spain reported that seasonal changes in their diet, with the consumption of richer foods in the autumn (fall) increased the prevalence of their AP episodes.

*“Maybe it's autumn, I do not know whether it is because of the change of food or what, because it's normally lighter in Summer, let's say that they are “gazpachitos” [cold tomato soup], more liquid things. When winter comes, we eat heavier food, I get [AP attacks] more often” *− 113 (F, 60 years, Spain, never on volanesorsen).

However, three participants said they had episodes even when they had been careful about their diet and these seemed to happen with no trigger. Other reported triggers included strenuous activity and stress.

#### Other abdominal pain

Thirteen participants reported experiencing abdominal pain separate to episodes of AP.

*“More often than not had a low level stomach ache” *− 110 (F, 58 years, UK, previously on volanesorsen).

Five participants said they would interpret this pain as a warning that they might have an episode of AP that could be treated with pain killers or by being even more cautious with their diet.

#### Gastrointestinal (GI) symptoms

All participants reported experiencing some GI symptoms, including diarrhea, constipation, indigestion, bloating or nausea.

*“This bloating doesn't let you move…You are completely, as I say, impaired.” –* 113 (F, 60 years, Spain, never on volanesorsen).

Two participants also reported having steatorrhea (oily stools), and one participant reported that they sometimes passed undigested food. Some participants reported that food was a trigger for these symptoms, but others did not know what caused them. These symptoms were generally reported as being fairly constant, but one participant said they worsened during menstruation.

#### Fatigue

All but one participant reported experiencing tiredness and fatigue, for most participants this was seen as a symptom of FCS, however one participant believed their fatigue was related to their age and occupation. Eight participants reported feeling constantly fatigued.

*“…It was continuous, continuous all the time, it could be anything, I’d go for a walk, you come back and you’re tired, you go out on a bike, you come back and you’re tired, you’re tired after every, you eat and then you’re tired…”* − 103 (F, 48 years, UK, currently on volanesorsen).

Fatigue and tiredness often coincided with other symptoms, for example, two participants said tiredness usually preceded an episode of AP, and another said they felt fatigued when they had GI issues. One participant described the tiredness they experienced after an episode of AP:

*“…Also the recovery post- pancreatitis, so people often, or work would often say, “You're out of hospital, when are you coming back?”, and actually there’s those weeks of recuperation still to happen, where you've lost so many calories, you've lost so much weight, you feel so physically tired that you can't actually do anything…”* – 104 (F, 32 years, UK, currently on volanesorsen).

#### Cognitive function

Eleven participants reported that FCS had an impact on their cognitive function, in particular memory and concentration. Some participants described experiencing brain ‘fog’. One described this as being confused when other people recalled events that they could not remember, and the other said they were unable to recall words or names.

*“It’s almost like a fog and you’re treading water inside trying to find the words that you want to say and like I say, forgetting names, forgetting words for things”* – 109 (F, 52 years, UK, previously on volanesorsen).

Another described being forgetful about what they were doing or losing focus and forgetting vocabulary during conversations.

Participants reported a range of triggers for their poor memory loss, attention, and concentration. One participant reported that their memory and attention were worse on days when they had not eaten much, and they needed to be reminded to take their medication. Another linked their issues with memory to stress and high levels of fat in their diet:

*“…My memory kind of gets worse and I find it harder to concentrate when I'm ... Whether that's to do with stress, or whether that's to do with high fats, I don't know.”* – 110 (F, 58 years, UK, previously on volanesorsen).

One participant with diabetes reported having trouble concentrating when their blood sugar levels were high.

### Impacts of FCS

#### Impact on daily activities/work

FCS had a large impact on the daily activities of participants in the study, particularly as a result of episodes of AP:

*“My life was interrupted very quickly and very profoundly. So whatever I was doing, whether it be studying or working or on holiday or whatever, my life could change very quickly, and I’d be hospitalized and have no control.” *− 01 (M, 41 years, UK, currently on volanesorsen).

Episodes of AP were reported as one of the biggest impacts on work, as they frequently involved extended periods of absence due to hospitalizations and subsequent recuperation. Two participants spoke of their concerns about missing work and felt they were letting their colleagues down:

*“I had time off work, every time I went back it was like starting a new job… sometimes I went back for a couple of my night shifts and then I ended up back off again because I'd gone back too early, so I don't like to let people down, so that affected me a lot” *− 103 (F, 48 years, UK, currently on volanesorsen).

Other participants described how repeated absences from work had made it difficult to get and maintain a job or meant they were overlooked for promotion.

*“I have been fired, because as soon as they know that you have a rare disease that has no cure, what they do is that they, they fire you from the jobs because they don't want people who might cause them problems.” *− 114 (F, 45 years, Spain, previously on volanesorsen).

As well as paid work, some participants described how episodes of AP impacted their ability to do housework, as they were too physically weak to even manage washing and drying up when they were discharged from hospital or after a day at work. Two participants reported an impact on their ability to travel as they were worried about being ill when away from home. Some participants described how it was difficult for them to exercise because of their FCS.

#### Impact on social activities

Many participants reported that FCS impacted their social lives and described how they would have to weigh up the pros and cons of each social occasion to decide if it was worth it.

*“…The whole condition played havoc with my social life…I would have to feel that what I was going out to do was going to be worth it”* − 110 (F, 58 years, UK, previously on volanesorsen).

Thirteen participants said that their dietary restrictions impacted their ability to socialize normally with friends and family. Several participants described how their FCS made it very difficult for them to socialize, as it was difficult to find activities that do not involve food or drink:

*“I really try and suggest things that aren't to do with food, and it’s so hard because most of social activities are food based or drink based”* − 104 (F, 32 years, UK, currently on volanesorsen).

When they did go out, participants reported finding it difficult to find something they could eat because their diet was so restricted. Some participants reported that they avoided social situations altogether, as the stress of worrying about what to eat or risking becoming unwell again made them not want to go out at all.

*“I don't attend baptisms, nor weddings, nor communions, nor do I get together with people for Christmas. I am alone, always at home.”* − 118 (F, 47 years, Spain, previously on volanesorsen).

#### Impact on family and relationships

Sixteen participants reported that FCS had an impact on their family and/or romantic relationships. One younger participant said it was difficult to date when they were frequently unwell and in and out of hospital:

*“You can't go out and date, and you just don’t have the energy, and also you don’t want to burden somebody new with a condition that was such a high burden for me at that time, so I think I was depressingly single as well which didn’t help how I felt about myself*” − 104 (F, 32 years, UK, currently on volanesorsen).

Others described how their FCS caused relationship issues, including a lack of understanding from their family, reduced sex drive, and arguments with their partner:

*“I'm tired all the time, obviously I just want to not do anything, but then it will affect my relationship because I'm not wanting to get up, obviously we’ll have an argument because I just want to stay in bed”* − 106 (M, 27 years, UK, never on volanesorsen).

One participant reported that FCS led to the breakdown of their relationship:

*“My first boyfriend, I lost him because he got bored, because I had pancreatitis episodes, I spent more time in hospital than walking around with him”* − 113 (F, 60 years, Spain, never on volanesorsen).

In contrast, another participant said their FCS had brought them closer to their spouse, who was very protective and understood the condition well.

Four participants reported having extremely complicated pregnancies with frequent stays in hospital. Two participants had an episode of AP while pregnant, which resulted in one participant deciding not to have another biological child.

### Management of FCS

All participants reported controlling their diets by restricting their fat intake considerably. They did this by eating foods that were low in fat and avoided cooking in oil.

Some of the food and drink participants reported eating included: rice, pasta, cereal, egg whites, fish, tuna in water, chicken, grilled lean meat, medium-chain triglyceride (MCT) oil, lentils, vegetables, potatoes, stews, salad, tomatoes, gazpacho, fruit, skimmed milk, yoghurt, toast with jam, water, tea, coffee, orange juice and tomato juice. The food participants avoided included: dairy, fried food, chocolate, biscuits, sweets, ice cream, pastry, eggs, sugar, fizzy drinks, alcohol, sauces, salad dressing, non-lean meat, chorizo, tapas, curry, and Chinese food.

Nine participants reported eating less food or fasting completely to manage FCS symptoms and avoid having an AP episode.

*“…If I haven’t been feeling well for a few days then I have a day when I just stick to water and drink water all day and that really helps sometimes to cut back on the pancreatitis attacks” *− 109 (F, 52 years, UK, previously on volanesorsen).

Participants were divided as to how satisfied they were with their diet. Seven said they did not enjoy their diet, either because it involves constant denial of certain foods or it was boring and tasteless, or they did not eat enough. Three described their diet as repetitive, boring and monotonous. One participant reported how they ate with fear which made the process less enjoyable. Those who were most satisfied with their diet were those with a preference for low-fat foods such as fruit and vegetables.

Participants reported several challenges of adhering to their diet and the social impact of not being able to eat out as described above. In addition, participants described the burden of having to constantly monitor their fat intake, lack of flexibility, needing to plan meals, difficulty storing food in the workplace, and being unable to eat the same food as their families.

*“I’m not the person I was because it controls my life, I’ve had no flexibility in it at all and the thought of something coming that potentially could help lower the lipids…was like amazing, except for just the diet”* − 109 (F, 52 years, UK, previously on volanesorsen).

Co-morbidities, such as diabetes made the diet even more difficult to manage.

*“I was diagnosed with diabetes about 10 years ago and I’m on insulin. So, to manage a diet that should be low in carbohydrate, low in fat, low in sugar is just unreal, it can’t be done, so it wasn’t a very nice life really. Just with me, I make the most of it, just cook well but psychologically it’s changed me as a person” *− 109 (F, 52 years, UK, previously on volanesorsen).

One participant described how they had been unable to find a dietician or nutritionist that was able to meet their needs, while another explained difficulty of finding food products that met dietary requirements. Support from patient associations or other family members with FCS, advice from a dietician, diet apps, or recipes on social media were reported as facilitators of participants’ diet management.

*“I log it all on my phone, so your phone overestimates it I think a little bit, but it would be about 12 to 15 gram a day, which is the same as what I was doing before the drug but I was obviously getting ill frequently even at that level before…one of the major drawbacks of [the app] is it’s very generic”* − 104 (F, 32 years, UK, currently on volanesorsen).

Several participants mentioned the role their close family members played in helping them to maintain a strict dietary regimen.

Of the participants who had received volanesorsen, several reported an improvement in their symptoms following treatment. This included an improvement in episodes of AP, abdominal pain, gastrointestinal symptoms, fatigue and cognitive function.

*“The pain was [comparatively] less, I must say, it was comparatively less. I stayed only for four days or something like that”* − 107 (F, 44 years, UK, currently on volanesorsen).

*“Being on [volanesorsen], for me, the truth, it has made me very happy, because I felt like more, more active, right? Not, not so tired, not so exhausted” *− 114 (F, 45 years, Spain, previously on volanesorsen)

Improvements in impacts were also reported, with some participants reporting that their daily activities and work, social activities, family and relationships, and psychological wellbeing.

*“It’s just improved every part of [life], to feeling physically well and not having to go to hospital for pain relief, and to be able to work” *− 104 (F, 32 years, UK, currently on volanesorsen).

Some participants also reported that their diet had improved since starting volanesorsen, as they were able to eat a little more fat without worrying and they felt more confident in choosing food.

*“I’d get up, have me diet, have my lunch and then have my tea but what it gave me were a little bit of flexibility or confidence. If somebody had made me a sandwich or something, to be able to eat that and think, “The [volanesorsen] will help” and it was great, absolutely great”* − 109 (F, 52 years, UK, previously on volanesorsen).

## Discussion

The symptoms and impacts of FCS identified in this study are consistent with a previous qualitative study with individuals with FCS that aimed to identify concepts to include in a new patient-reported outcome measure. This included concerns about pancreatitis attacks, adherence to a restrictive diet and the social limitations associated with that, future health, mobility, depression, financial impact and productivity [13]. However, this is the first study to present the findings in a conceptual model to illustrate the relationships between the symptoms and impacts. The patient-centered conceptual model was developed based directly on the concepts reported by participants in these interviews. Given the relationships between the concepts illustrated in the conceptual model, these findings show the potential impact of a treatment to extend beyond core symptoms to other aspects of quality of life. For example, a reduction in episodes of AP, may result in an improvement in abdominal pain, gastrointestinal symptoms and fatigue, which in turn may improve daily activities and work, social activities, family and relationships, and psychological wellbeing. As well as illustrating the relationships between concepts, the conceptual model can be used as a tool for to inform the selection and content validation of patient-reported outcome instruments in clinical trials of the evaluation of new treatments for FCS.

The findings of this study highlight that episodes of AP were by far the most impactful aspect of living with FCS. These were associated with a range of symptoms, most notably extremely severe abdominal pain, but also nausea and vomiting, fever, bloating and appetite loss. All but one participant had experienced AP at some point in their lives with varying frequency, however, several participants reported improvements in pain during episodes of AP following treatment with volanesorsen. Other symptoms and functional issues associated with FCS included abdominal pain separate to an AP episode, gastrointestinal symptoms, issues with cognitive function, and fatigue.

The second most impactful aspect of living with FCS was following a low-fat diet. The main reason for this was to avoid an episode of AP. Adhering to their diets severely restricted their lives, this led to them avoiding social situations and other events, which in turn made it difficult for them to build and maintain relationships with partners, friends, and family.

Episodes of AP typically involved individuals being hospitalized for a period of days or weeks, as well as having a long recovery period, which meant they were often off work for long periods of time. This made it difficult for some to maintain a job or complete their studies. Food and alcohol were reported as triggers for episodes of AP. Some participants described how their FCS impacted their relationships with their partners or made it difficult for them to date or had impacted their ability to have children. These symptoms and impacts had a substantial influence on their psychological wellbeing. This is consistent with previous findings assessing the impacts of FCS on individual’s lives, where FCS was found to affect employment, emotional wellbeing, and social relationships [3]. Furthermore this study provided novel insights on the impact of treatment with volanesorsen. Although improvements cannot be attributed to volanesorsen based on this study, they provide some unique perspectives on the experience of treatment.

While this study provides novel insights, it also had some limitations that need to be acknowledged. Most (N = 13) participants were currently or had previously been treated with volanesorsen. While this was necessary for exploring the impact of volanesorsen, the study could also have included more individuals who had never been treated with volanesorsen, as those eligible for treatment may have different characteristics to those who are not eligible. In addition, most participants therefore had to rely on retrospective recall when asked about their experience of FCS before they started treatment. However, four participants who had never been treated did not report notable differences in their experiences, compared with those who had been treated. Finally, although qualitative research is not designed to be representative, it is possible that individuals recruited from patient advocacy groups may differ from those who do not join such groups. This may partly explain why a larger number of women took part in the study. However, we also recruited from clinical sites in Spain, which should minimize the impact of this.

## Conclusions

This qualitative study reports on the patient experience of symptoms and impacts of FCS and outlines the relationships of such symptoms and impacts in a conceptual model. Findings highlight the substantial burden of FCS, and the restrictions individuals face in their management of their symptoms and the potential of treatments to address this high unmet need by improving symptoms and HRQoL and reducing clinical events in this patient population.

## Data Availability

Raw data (interview transcripts) are not publicly available to protect participant privacy, as FCS is a rare disease and there are very few participants in each country.
